# Comparative analysis of cancer genes in the human and chimpanzee genomes

**DOI:** 10.1186/1471-2164-7-15

**Published:** 2006-01-26

**Authors:** Xose S Puente, Gloria Velasco, Ana Gutiérrez-Fernández, Jaume Bertranpetit, Mary-Claire King, Carlos López-Otín

**Affiliations:** 1 Departamento de Bioquímica y Biología Molecular, Facultad de Medicina, Instituto Universitario de Oncología, Universidad de Oviedo, 33006-Oviedo, Spain; 2 Unitat de Biologia Evolutiva, Departament de Ciencies Experimentals i de la Salut, Universitat Pompeu Fabra, Barcelona, Spain; 3 Departments of Medicine and Genome Sciences, School of Medicine, University of Washington, Seattle WA-98195, USA

## Abstract

**Background:**

Cancer is a major medical problem in modern societies. However, the incidence of this disease in non-human primates is very low. To study whether genetic differences between human and chimpanzee could contribute to their distinct cancer susceptibility, we have examined in the chimpanzee genome the orthologous genes of a set of 333 human cancer genes.

**Results:**

This analysis has revealed that all examined human cancer genes are present in chimpanzee, contain intact open reading frames and show a high degree of conservation between both species. However, detailed analysis of this set of genes has shown some differences in genes of special relevance for human cancer. Thus, the chimpanzee gene encoding p53 contains a Pro residue at codon 72, while this codon is polymorphic in humans and can code for Arg or Pro, generating isoforms with different ability to induce apoptosis or interact with p73. Moreover, sequencing of the *BRCA1 *gene has shown an 8 Kb deletion in the chimpanzee sequence that prematurely truncates the co-regulated *NBR2 *gene.

**Conclusion:**

These data suggest that small differences in cancer genes, as those found in tumor suppressor genes, might influence the differences in cancer susceptibility between human and chimpanzee. Nevertheless, further analysis will be required to determine the exact contribution of the genetic changes identified in this study to the different cancer incidence in non-human primates.

## Background

Cancer is a major and growing clinical problem in modern societies. Although usually referred to as a single disease, cancer represents more than 200 different pathologies, which are characterized by an uncontrolled cell growth that may derive in the invasion of surrounding tissues and the subsequent generation of metastasis in distant organs of the body [1,2]. Tumor development is a complex process in which genetic, epigenetic and environmental factors are implicated [3-6]. The importance of genetic factors in cancer is now well established, as mutations in specific genes have been associated with the neoplastic transformation and development of specific cancer types [3,7,8]. This fact is further supported by the existence of hereditary cancer syndromes, caused by germ-line mutations in specific genes and responsible for about 5% of all diagnosed malignancies [9-11]. Over the last two decades, a number of studies have focused on the identification of the different genes that can contribute to cancer. These studies have led to the conclusion that alterations in three types of genes – oncogenes, tumor-suppressor genes, and stability genes – are mainly associated with the genesis of cancer [3]. These studies have alsocontributed to elucidate the molecular mechanisms through which these genes act during tumor development and progression [12]. Finally, in some cases this knowledge has resulted in the introduction of new therapeutic strategies for cancer treatment [13-16].

Chimpanzees (*Pan troglodytes*) represent our most closely related organism. They have a more similar physiology to human than any other model organism, and the study of several human diseases in chimpanzee has led to a better understanding of pathologies such as hepatitis or AIDS [17,18]. Interestingly, a number of works have reported that cancer incidence in non-human primates is very low. This fact is especially evident for epithelial neoplasms such as breast, prostate or lung carcinomas, which are responsible for more than 20% of human deaths but whose incidence in great apes is less than 2% [18-22]. The difference in cancer incidence between human and chimpanzees can mainly derive from three facts: i) exposure to different environmental factors, including diet and habits, ii) differences in life expectancy and iii) genetic differences that might result in humans being more susceptible to cancer development.

The completion of the first draft of the chimpanzee genome sequence has opened the possibility to study whether genetic differences between human and chimpanzee could contribute to the observed differences in cancer susceptibility between both species [23]. To address this question, we have used the chimpanzee genome sequence to identify and compare the orthologous genes of a set of 333 human genes that have been previously implicated in cancer development [3,7]. This analysis has revealed that all analyzed human cancer genes are present in the chimpanzee and exhibit a high degree of conservation between both species.

However, further detailed analysis of a series of genes of special interest in human cancer such as *p53 *and *BRCA1*, has revealed some differences with the corresponding chimpanzee counterparts. In this work, we present the results of this comparative genomic analysis and discuss the putative relevance of the observed genetic changes for explaining some aspects of the differential cancer susceptibility between human and chimpanzee.

## Results

### Analysis of cancer genes in the chimpanzee genome

**Figure 1:**
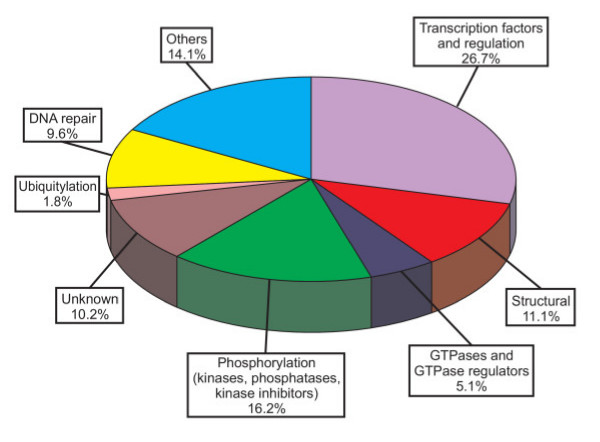
**Distribution of cancer genes in different functional categories**. The percentage of genes present in each category is indicated.

As an initial attempt to study cancer-associated genes in the chimpanzee, we have compared 333 human cancer genes to the chimpanzee genome and identified and analyzed the orthologous genes (see Additional data file 1). The set of human cancer genes was selected from the literature based on mutational analysis and/or roles in processes such as chromosomal stability, promoter methylation or control of mitotic checkpoints [3,4,7,24-28]. As can be seen in Figure 1, more than 50% of the genes belong to three main functional categories: transcription factors, phosphorylation (including kinases, kinase inhibitors and phosphatases), and DNA repair. Other functional groups include structural proteins, GTPases and GTPase regulators or proteins involved in ubiquitylation. Finally, other minor groups include tumor suppressors, the protein and RNA components of the telomerase, or proteins implicated in apoptosis among others.

To perform this study, the cDNA sequence from all human cancer genes was first compared with the chimpanzee genome in order to determine the presence or absence of the corresponding orthologs, and then to predict their complete cDNA sequences. This analysis revealed that all analyzed human cancer genes are present in the chimpanzee genome. The high sequence coverage of this set of cancer genes (96% coverage at the nucleotide level, 1,161,044 nucleotides), has allowed us to perform a detailed comparison – at the nucleotide and amino acid level – of cancer genes between both species (see Additional data file 1 for more details).

Direct comparison of human and chimpanzee cancer genes indicates that they are highly conserved, showing 99.38% identities at the protein level, and 99.19% at the nucleotide level, what is similar to the average amino acid identities between both organisms (99.38%) [23]. Interestingly, we have identified 71 cancer genes (representing 21% of the total) encoding proteins which are 100% identical to their human counterparts (Additional data file 2). The conservation of cancer genes between human and chimpanzee suggests that they perform essential cellular functions and are in agreement with previous studies [29]. This proposal is consistent with the fact that most analyzed cancer genes encode intracellular proteins highly conserved from yeast to human, and implicated in fundamental processes such as cell signalling, cell cycle control and maintenance of genomic stability [3].

### Analysis of insertions and deletions in protein coding regions from thechimpanzee genome

Despite the high conservation between human and chimpanzee cancer genes described above, in the course of the present study we identified 20 chimpanzee genes (6% of all analyzed genes) that encode proteins containing or lacking specific residues due to the insertion or deletion of codons in the corresponding open reading frames (Table 1). The analysis of these genes can be important in order to identify human-specific changes in protein coding genes. A total of 9 chimpanzee genes encode proteins containing extra amino acids in their sequence, 6 of them located within trinucleotide repeats resulting in longer expansions in chimpanzee, with up to 4 extra glutamine residues in the case of *MN1*. On the other hand, 12 chimpanzee genes encode proteins with less amino acids than their human counterparts. Similarly to the expanded proteins in chimpanzee, 8 of the 12 expansions in human proteins were located in trinucleotide repeats. The higher proportion of longer alleles in humans than in chimpanzee is consistent with known results [30]. Interestingly, analysis of these expanded regions using information retrieved from EST databases, resulted in the finding that six human genes, *ASPSCR1*, *DEK*, *FANCC*, *MLLT3*, *MYST4 *and *ZNF384*, are polymorphic for these loci. As functional trinucleotide tandem repeats that vary in humans have been shown to be also variable in chimpanzee [30], it is likely that all these genes present variability in both species. These results suggest that these regions might be prone to genetic instability, and open the possibility to investigate if all these genes showing human-chimpanzee variability in trinucleotide repeats are polymorphic in humans and influence the risk of tumor development.

During the course of this analysis, we also observed that 52 genes (representing 15.6% of all analyzed cancer genes) contained putative frameshifts and/or premature stop codons in the coding sequences (Additional data file 3). This high number of conflictive regions is not likely to reflect *bona fide *differences between these species, but the result of sequencing problems or artefacts derived from the assembly process. To evaluate these possibilities, we took advantage of the availability of two independent assemblies for the chimpanzee genome, called ARACHNE and PCAP [23]. The ARACHNE assembly, containing more coverage and fewer order conflicts, is used by most public genome browsers and was employed in the initial steps of this study. The PCAP assembly is a *de novo *assembly that is non-redundant with ARACHNE and provides an additional resource for this kind of sequencing conflicts. To distinguish artefacts of the assembly process from real differences or sequencing errors, we compared the conflictive regions identified in cancer genes to the PCAP assembly. This strategy resulted in the resolution of 83% of the conflicts, as they were correct in the PCAP assembly, thereby suggesting that they represented errors generated during the assembly process. Nevertheless, 9 cancer genes (*BCL3*, *ELL*, *GAS7*, *MLL*, *MLLT3*, *NF1*, *RECQL4*, *SMARCB1 *and *TPR*) still contained the same frameshift in both assemblies. Because these genes might represent real differences between human and chimpanzee cancer genes, we PCR-amplified all these conflictive regions, and the resulting DNA fragments were subjected to direct sequencing. This strategy allowed us to confirm that these genes do not contain frameshifts or premature stop codons, and encode functional proteins in all cases. These findings call the attention to the degree of accuracy of the chimpanzee genome despite the existence of two assemblies. Ongoing efforts to increase the shotgun coverage from ~4 fold to ~8 fold redundancy will produce a higher quality sequence necessary for a reliable ascertainment of specific interesting discrepancies.

### Analysis of missense mutations in cancer genes in the chimpanzee genome

Mutations in human cancer genes can be originated by different causes, including chromosomal translocations resulting in fusion proteins, disruption of the coding sequence by frameshifts or premature stop codons, and point mutations that modify the structure and function of the corresponding protein. As mentioned above, we did not find evidence in the chimpanzee genome of gene fusions, frameshifts or premature stop codons in the set of analyzed cancer genes. However, it is interesting to study the status in the chimpanzee genome of residues that are variable in human genes and are associated with cancer. For this purpose, we extracted the information of variant alleles in human genes from the Human Gene Mutation Database (HGMD) and the Online Mendelian Inheritance in Man (OMIM) databases [31,32], and determined the corresponding residue in the chimpanzee protein. We found two genes associated with breast cancer, *BRCA2 *and *ERBB2*, in which the chimpanzee sequence differed from the human in residues that have been reported to be polymorphic in humans. Both alleles, *BRCA2 *(N372H) and *ERBB2 *(V655I), have been associated with different risk of developing breast cancer [33,34]. Interestingly, in both cases the chimpanzee sequence (N372 and I655) corresponds to the allele that has been associated with a reduced risk of cancer, and both are being found at high frequencies in humans, around 0.8 for N372 [34] and from 0.7 to 0.9 for I665.

We also identified at least two cases in genes associated with colon carcinomas, *MLH1 *(A441T) and *MSH2 *(S323C), and one with prostate carcinomas, PON1 (I102V), in which the susceptibility allele in human appears to be the wild-type allele in chimpanzee. However, due to the limited clinical and functional data on these variants, the biological significance of these findings is still unknown, and further studies will be required to determine the pathogenic activity of these alleles.

### Analysis of the tumor suppressor gene *p53 *in the chimpanzee genome

A detailed analysis of the gene encoding the tumor suppressor p53, which is the most frequently mutated gene in human cancer [35], showed a single amino acid difference between human (Arg at position 72) and chimpanzee (Pro72). Interestingly, this p53 codon is polymorphic in human and the allele encoding Pro72 is frequent in some populations [36]. Analysis of chimpanzee DNA from four different geographic regions revealed a Pro/Pro genotype in all cases, what confirmed our findings using the chimpanzee genomic sequence. However, we cannot rule out the possibility that this codon might be polymorphic in chimpanzee and that the Arg72 allele could be present in some individuals, what would require the analysis of a greater number of samples. In this sense, sequencing of 14 chimpanzee samples revealed a Pro/Pro genotype, suggesting that this allele would be the most common one in chimpanzee, with a Poisson distribution maximum estimate of allele frequency of 0.1 at a 95% confidence interval.

**Figure 2:**
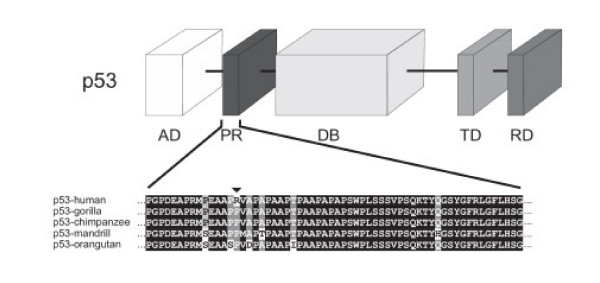
**Amino acid alignment of the p53 proline-rich domain from different primates**. Schematic representation of p53 protein (AD: activator domain, PR: proline-rich domain; DB: DNA-binding domain; TD: tetramerization domain; and RD: C-terminal regulatory domain). The proline-rich domain containing Arg72 (*arrowhead*) was sequenced in different primate species and aligned to the human sequence.

Additionally, we have sequenced the same p53 region in different primates, including gorilla, orangutan or mandrill, and determined that codon 72 codes for Pro in all species (Fig. 2). The fact that apes and some Old World monkeys contain Pro at this position, suggests that it is the ancestral allele and the Arg72 allele is unique to the human lineage. Nevertheless, the ancestral Pro/Pro genotype is still present in humans, representing about 8.3% of the Central-European population and 45% of the African yoruba population (see dbSNP rs1042522) [37]. This finding raises interesting questions about the different susceptibility to cancer of both organisms, as several studies have shown that both isoforms are functionally different in their ability to induce apoptosis or interact with p73 [38-40].

### Analysis of the *BRCA1 *locus in the chimpanzee genome

The tumor suppressor *BRCA1 *has an unusual evolutionary history, in that ratios of replacement to silent nucleotide substitutions (K_A_/K_S_) are greater than one when comparing human and chimpanzee lineages, but not in lineages of other primates or other mammals [41-43]. This observation is consistent with positive selection pressure on *BRCA1 *during recent hominoid evolution. We evaluated the complete human and chimpanzee genomic sequences of *BRCA1 *in light of this observation. In human and chimpanzee, the *BRCA1 *locus spans ~130 kb, including the *BRCA1 *gene itself and a partially duplicated 5' (telomeric) region. The duplication includes the highly conserved bidirectional promoter regulating *BRCA1 *and the adjacent gene *NBR2*, a *BRCA1 *partial pseudogene, and *NBR1/M17S2*. The number of nucleotide differences between human and chimpanzee sequences at the *BRCA1 *locus is not remarkable, 1.15%, and does not vary appreciably between the *BRCA1 *locus and the duplicated region. However, humans and chimpanzee sequences differ by an 8 Kb insertion/deletion within the partially duplicated region. The deletion on the chimpanzee sequence prematurely truncates the *NBR2 *gene. The consequences to *BRCA1 *expression in chimpanzee of the truncation of the adjacent, co-regulated gene are not known. In mouse, the region is not duplicated and the bidirectional promoter co-regulates *Brca1 *and *Nbr1 *[44].

## Discussion

The availability of the chimpanzee genome sequence provides an excellent opportunity to explore the genetic basis for some of the biological differences between human and our most closely related organism [23]. An striking finding in this regard is the observation that non-human primates show a lower incidence of cancer than humans, specifically for epithelial carcinomas [18,20]. To investigate if this different susceptibility to cancer was due to genetic differences between humans and chimpanzees, we performed a comparative analysis in the chimpanzee genome of a set of 333 human genes that have been directly implicated in tumor development [3,7]. This analysis revealed that all human cancer genes contain a clear ortholog in the chimpanzee genome, and show a percentage of identities at the protein level similar to the genome average (99.38%). Nevertheless, the strict conservation of this group of more than 330 genes contrasts with the recent analysis of other groups of genes, including a set of 560 proteases, which shows that at least 7 genes are absent in either human or chimpanzee genomes [45], or the CD33-related Siglecs, which also show specific loss of some genes in the human lineage [46]. These data suggest that despite cancer genes show a percentage of identities similar to the genome average, they are perfectly conserved between human and chimpanzee, confirming previous studies showing a higher conservation of genes implicated in essential cellular functions [29].

This study has shown that despite the high degree of identity in cancer genes, there are 1542 amino acid changes whose contribution to the different cancer susceptibility between human and chimpanzee should be further investigated. Nevertheless, additional factors contributing to the observed differences could include changes in diet, lifestyle or exposure to mutagenic agents [47-49]; physiological differences in immune system or in life expectancy and aging rates [50]; variations in gene expression, alternative splicing or in DNA methylation patterns [51,52]; or alterations in other genes not analyzed in this study. In this regard, we must emphasize that we have compiled a group of 333 genes that are causally implicated in cancer development as a result of mutational analysis or owing to their participation in processes such as control of mitotic checkpoints, chromosomal stability or promoter methylation. This set of genes includes those annotated in a recent census of human cancer genes [7], but also incorporates novel genes described to be mutated in human carcinomas [3,4,25-28]. However, the possibility that other yet unknown cancer-related genes are responsible for the different susceptibility to this disease between human and chimpanzee, cannot be ruled out.

In relation to the existence of functional differences in the immune system of human and non-human primates, it is now clear that the immune and inflammatory responses play important roles in cancer development and progression [53-56]. Accordingly, structural and functional variations in genes associated with these processes might be responsible for the species-specific susceptibility to certain tumors. Life expectancy is another factor that might contribute to the observed differences in cancer incidence between human and chimpanzee. Although age is known to represent a major risk factor for cancer development, the observation that non-human primates have a lower incidence of epithelial carcinomas but not other cancers [18], suggests that life expectancy might contribute only partially to the observed differences in cancer susceptibility. On the other hand, and despite the strong conservation in the coding regions of cancer genes from human and chimpanzee, it is possible that differences in regulatory elements outside these coding regions can result in changes in the expression levels or in the tissue distribution patterns [57]. In fact, recent reports have shown considerable differences in the expression levels of orthologous genes from different primate species [58-60], supporting the idea that regulatory changes might account for some interspecies differences, including cancer susceptibility [61].

Despite the high conservation of cancer genes between both species, we identified 20 genes containing several codon insertions or deletions in their protein coding regions, although the functional significance of these differences, including their putative association with cancer, will require further studies. It is interesting to note that in 70% of these cases, the insertion/deletion event occurred within trinucleotide repeats, suggesting that these regions could be prone to genetic instability, in both human and chimpanzee genomes. Analysis of EST databases allowed us to confirm this hypothesis, as six human genes, *ASPSCR1*, *DEK*, *FANCC*, *MLLT3*, *MYST4 *and *ZNF384*, were polymorphic in the number of repeats in these regions. In the case of genes implicated in cancer, it will also be interesting to study if some of the identified haplotypes could confer a higher risk of tumor development.

The fact that most cancer genes show a high degree of conservation between human and chimpanzee, prompted us to analyze in more detail the observed changes in genes previously reported to be of special relevance in human cancer, such as the tumor suppressors *p53 *and *BRCA1*. We found that chimpanzee p53 shows a single amino acid difference with human p53, resulting in a protein containing Pro instead of Arg at position 72. Interestingly, this codon is polymorphic in human, and the Pro72 residue is common in all studied human populations [36,62]. Sequencing of this region in other non-human primates allowed us to confirm that this Pro residue has been conserved during evolution, and suggests that the Arg72 allele, present in 55–92% of the human population, arose in the human lineage. The presence of Pro72 in chimpanzee might have physiological consequences, as several reports have found interesting functional differences between both p53 isoforms. In fact, p53-Arg72 protein has an increased ability to induce apoptosis, to translocate from the nucleus to the mitochondria, to be degraded by the E6 oncoprotein of human papillomavirus or to inactivate p73 when mutated [38-40]. Different studies have provided evidence for an increasedrisk of cancer development associated with the Arg72 genotype, although this topic has been controversial and further studies will be necessary to definitely address this question [39,40,63]. Therefore, and although the presence of Pro72 in chimpanzee p53 might contribute to the reduced cancer susceptibility in non-human primates, further work will be required to confirm this hypothesis.

Analysis of the tumor suppressor *BRCA1 *has shown that it has been subjected to positive selection during recent hominoid evolution, as the overall K_A_/K_S _ratios are greater than one when comparing human and chimpanzee lineages, but not on lineages of other primates or mammals, as has been recently shown [43]. Additionally, the chimpanzee BRCA1 locus contains an 8 Kb deletion in the partially duplicated 5' region. This deletion prematurely truncates the *NBR2 *gene, which is regulated by a bi-directional promoter that co-regulates *NBR2 *and *BRCA1*. The consequences to *BRCA1 *expression in chimpanzee of the truncation of the adjacent, co-regulated gene are not known. The distinctive evolution of human and chimpanzee *BRCA1 *suggest that an evolutionary approach may be important to understanding selection at this, and perhaps other cancer associated genes [43,64,65].

## Conclusion

In summary, in this work we have performed an analysis of a defined set of 333 genes to try to elucidate the molecular basis of human-chimpanzee differences in one aspect of significant biomedical relevance: the interspecies variability in cancer susceptibility. The overall picture emerging from this comparative analysis is one that reflects the high degree of conservation in this group of cancer genes, although specific differences in relevant genes such as *p53 *and *BRCA1 *can illuminate new functional and evolutionary aspects of these tumor-suppressor genes. Altogether, the limited genetic variability found in this human-chimpanzee comparative analysis might contribute to the different cancer susceptibility between these closely related species. However, further investigations will be necessary to determine the influence of these genetic changes in cancer, or whether additional factors, such as changes in gene regulation, immune system genes, life expectancy or environmental influences, might also contribute to this process.

## Methods

### Bioinformatic screening of the chimpanzee genome

A database of human cancer genes was constructed by using information from the literature, and the corresponding cDNA and protein sequences were retrieved from the GenBank database. Each single human cDNA sequence was compared against the ARACHNE and PCAP chimpanzee genome assemblies by using a combination of BLAT and BLAST algorithms [66,67]. The corresponding chimpanzee cDNA and protein sequences were extracted and compared to the human ortholog by using the EMBOSS sequence analysis package. The full list of cancer genes, accession numbers and comparison to the human orthologs are available at in the Additional data file 1. In all cases, the primary chimpanzee cDNA sequence was obtained from the ARACHNE assembly, as this sequence is used by the Chimpanzee Genome Sequencing Consortium and by most public genome browsers (NBCI, Ensembl, or UCSC). However, in those cases where the chimpanzee gene was incomplete, the PCAP assembly was used to fill gaps in the genome, and the corresponding sequence was incorporated to the chimpanzee cDNA sequence for our analysis.

Conflictive regions were defined as those presenting frameshifts or premature stop codons in the chimpanzee coding sequence. Those regions were carefully examined in both ARACHNE and PCAP assemblies, as well as the corresponding human region in the human genome sequence and EST databases. In four cases (*LMO2*, *MLLT7, MN1*, and *MYST4*) the presence of a putative frameshift or premature stop codon in the chimpanzee sequence resulted from incorrect human sequence entries that contained frameshifts in the deposited entry. Modification of the human cDNA with the aid of EST and genomic sequences resulted in curated human cDNA and protein sequences, and the absence of frameshift or premature stop codon in the corresponding chimpanzee prediction. All other conflictive regions in the chimpanzee genome were solved by PCR amplification and direct sequencing of the corresponding gene using chimpanzee genomic DNA.

### PCR amplification and direct sequencing of chimpanzee genes

Chimpanzee, gorilla, orangutan and mandrill genomic DNA from different geographic regions was obtained using standard phenol-chloroform procedures. To analyze conflictive regions in the chimpanzee genome, we designed specific oligonucleotides flanking the exon of interest (Additional data file 3), and the corresponding region was PCR amplified from chimpanzee total genomic DNA in a Perkin Elmer 9700 thermocycler using High Fidelity Taq DNA polymerase or GC-RICH PCR system for regions rich in GC content (Roche Diagnostics). The amplified product was purified and subjected to automatic sequencing using the 5' oligonucleotide as primer using an ABI Prism 310 DNA sequencer (Applied Biosystems).

## Authors' contributions

XSP conceived this project, participated in the design and bioinformatic analysis, and drafted the manuscript. CLO also conceived this project and participated in the elaboration of the gene census. GV and AGF performed the bioinformatic analysis, gene comparison and PCR amplification and sequencing. MCK performed the analysis of the *BRCA1 *locus. JB provided DNA samples from different primates and helped with the analysis of p53. All authors read and approved the final manuscript.

## Supplementary Material

Additional data file 1 **Human and chimpanzee cancer genes comparison**. This file contains the complete list of human and chimpanzee cancer genes, and comparison at the nucleotide and protein level. Click here for file

Additional data file 2 **Chimpanzee cancer gene products identical to human orthologs**. List of human genes identical to chimpanzee orthologs. Gene name, accession number, chromosomal location, protein length and functional category are indicated. Click here for file

Additional data file 3 **Frameshifts in chimpanzee cancer genes**. List of chimpanzee cancer genes for which frameshifts or premature stop codons are present in the ARACHNE assembly, as well as the correct sequence obtained by direct sequencing or comparison with the PCAP assembly. Click here for file

## Figures and Tables

**Table 1:** Cancer genes with codons inserted/deleted in chimpanzee. Summary of chimpanzee cancer genes showing inserted/deleted codons with respect to the human sequence. The number of inserted/deleted codons, the amino acid change and whether the insertion/deletion occurred in trinucleotide expanded regions is shown. The status of this codon in human is also indicated.

**Gene**	**Codons**	**Residues inserted**	**Human status**
*Codons inserted in chimpanzee*			

CCNE1	1	S	-
FANCC	1	Q (in polyQ repeat)	Polymorphic
FGFR1	1	D (in polyD repeat).	-
HOXD11	1	G (in polyG repeat)	-
HSPCA1	1	E (in polyE repeat)	-
MLLT2	1	S	-
MLLT3	2	SS (in polyS repeat)	Polymorphic
MN1	4+1	QQQQ and Q (in polyQ repeats)	-
PML	2	GF	-

*Codons inserted in human*			

AF15Q14	1	R	-
ASPSCR1	2	ER (in polyER repeat)	Polymorphic
ATF1	1	A	-
DEK	1	E (in polyE repeat)	Polymorphic
DING	1+2	L and RD (in polyRD repeat)	-
EP300	3	MQQ (2 repeats in human, 1 in chimpanzee)	-
LMO2	1	G (in polyG repeat)	-
MLLT2	1	S (in polyS repeat)	-
MYST4	2+1	EE and E (in polyE repeats)	Polymorphic
NUMA1	4	AAIG	-
SMO	1	L (in polyL repeat)	-
ZNF384	1	Q (in polyQ repeat)	Polymorphic

## References

[B1] Hahn WC, Weinberg RA (2002). Modelling the molecular circuitry of cancer. Nat Rev Cancer.

[B2] Pantel K, Brakenhoff RH (2004). Dissecting the metastatic cascade. Nat Rev Cancer.

[B3] Vogelstein B, Kinzler KW (2004). Cancer genes and the pathways they control. Nat Med.

[B4] Feinberg AP, Tycko B (2004). The history of cancer epigenetics. Nat Rev Cancer.

[B5] Thilly WG (2003). Have environmental mutagens caused oncomutations in people?. Nat Genet.

[B6] Borgono CA, Diamandis EP (2004). The emerging roles of human tissue kallikreins in cancer. Nat Rev Cancer.

[B7] Futreal PA, Coin L, Marshall M, Down T, Hubbard T, Wooster R, Rahman N, Stratton MR (2004). A census of human cancer genes. Nat Rev Cancer.

[B8] Sugimoto M, Tahara H, Ide T, Furuichi Y (2004). Steps involved in immortalization and tumorigenesis in human B-lymphoblastoid cell lines transformed by Epstein-Barr virus. Cancer Res.

[B9] King MC, Marks JH, Mandell JB (2003). Breast and ovarian cancer risks due to inherited mutations in BRCA1 and BRCA2. Science.

[B10] Umar A, Risinger JI, Hawk ET, Barrett JC (2004). Testing guidelines for hereditary non-polyposis colorectal cancer. Nat Rev Cancer.

[B11] Lerman C, Shields AE (2004). Genetic testing for cancer susceptibility: the promise and the pitfalls. Nat Rev Cancer.

[B12] Hanahan D, Weinberg RA (2000). The hallmarks of cancer. Cell.

[B13] Druker BJ, Talpaz M, Resta DJ, Peng B, Buchdunger E, Ford JM, Lydon NB, Kantarjian H, Capdeville R, Ohno-Jones S, Sawyers CL (2001). Efficacy and safety of a specific inhibitor of the BCR-ABL tyrosine kinase in chronic myeloid leukemia. N Engl J Med.

[B14] Ross JS, Fletcher JA, Bloom KJ, Linette GP, Stec J, Symmans WF, Pusztai L, Hortobagyi GN (2004). Targeted therapy in breast cancer: the HER-2/neu gene and protein. Mol Cell Proteomics.

[B15] Adams J (2004). The proteasome: a suitable antineoplastic target. Nat Rev Cancer.

[B16] Lynch TJ, Bell DW, Sordella R, Gurubhagavatula S, Okimoto RA, Brannigan BW, Harris PL, Haserlat SM, Supko JG, Haluska FG, Louis DN, Christiani DC, Settleman J, Haber DA (2004). Activating mutations in the epidermal growth factor receptor underlying responsiveness of non-small-cell lung cancer to gefitinib. N Engl J Med.

[B17] Lanford RE, Bigger C, Bassett S, Klimpel G (2001). The chimpanzee model of hepatitis C virus infections. Ilar J.

[B18] Varki A (2000). A chimpanzee genome project is a biomedical imperative. Genome Res.

[B19] Waters DJ, Sakr WA, Hayden DW, Lang CM, McKinney L, Murphy GP, Radinsky R, Ramoner R, Richardson RC, Tindall DJ (1998). Workgroup 4: spontaneous prostate carcinoma in dogs and nonhuman primates. Prostate.

[B20] Beniashvili DS (1989). An overview of the world literature on spontaneous tumors in nonhuman primates. J Med Primatol.

[B21] McClure HM (1973). Tumors in nonhuman primates: observations during a six-year period in the Yerkes primate center colony. Am J Phys Anthropol.

[B22] Seibold HR, Wolf RH (1973). Neoplasms and proliferative lesions in 1065 nonhuman primate necropsies. Lab Anim Sci.

[B23] Mikkelsen TS, Hillier LW, Eichler EE, Zody MC, Jaffe DB, Yang S, Enard W, Hellmann I, Lindblad-Toh K, Altheide TK, Archidiacono N, Bork P, Butler J, Chang JL, Cheng Z, Chinwalla AT, deJong P, Delehaunty KD, Fronick CC, Fulton LL, Gilad Y, Glusman G, Gnerre S, Graves TA, Hayakawa T, Hayden KE, Huang X, Ji H, Kent WJ, King MC, KulbokasIII EJ, Lee MK, Liu G, López-Otín C, Makova KD, Man O, Mardis ER, Mauceli E, Miner TL, Nash WE, Nelson JO, Pääbo S, Patterson NJ, Pohl CS, Pollard KS, Prüfer K, Puente XS, Reich D, Rocchi M, Rosenbloom K, Ruvolo M, Richter DJ, Schaffner SF, Smit AFA, Smith SM, Suyama M, Taylor T, Torrents D, Tuzun E, Varki A, Velasco G, Ventura M, Wallis JW, Wendl MC, Wilson RK, Lander ES, Waterston RH (2005). Initial sequence of the chimpanzee genome and comparison with the human genome. Nature.

[B24] Zhou BB, Elledge SJ (2000). The DNA damage response: putting checkpoints in perspective. Nature.

[B25] Maser RS, DePinho RA (2002). Connecting chromosomes, crisis, and cancer. Science.

[B26] Rajagopalan H, Jallepalli PV, Rago C, Velculescu VE, Kinzler KW, Vogelstein B, Lengauer C (2004). Inactivation of hCDC4 can cause chromosomal instability. Nature.

[B27] Wang Z, Shen D, Parsons DW, Bardelli A, Sager J, Szabo S, Ptak J, Silliman N, Peters BA, van der Heijden MS, Parmigiani G, Yan H, Wang TL, Riggins G, Powell SM, Willson JK, Markowitz S, Kinzler KW, Vogelstein B, Velculescu VE (2004). Mutational analysis of the tyrosine phosphatome in colorectal cancers. Science.

[B28] Wang Z, Cummins JM, Shen D, Cahill DP, Jallepalli PV, Wang TL, Parsons DW, Traverso G, Awad M, Silliman N, Ptak J, Szabo S, Willson JK, Markowitz SD, Goldberg ML, Karess R, Kinzler KW, Vogelstein B, Velculescu VE, Lengauer C (2004). Three classes of genes mutated in colorectal cancers with chromosomal instability. Cancer Res.

[B29] Thomas MA, Weston B, Joseph M, Wu W, Nekrutenko A, Tonellato PJ (2003). Evolutionary dynamics of oncogenes and tumor suppressor genes: higher intensities of purifying selection than other genes. Mol Biol Evol.

[B30] Andrés AM, Soldevila M, Lao O, Volpini V, Saitou N, Jacobs HT, Hayasaka I, Calafell F, Bertranpetit J (2004). Comparative genetics of functional trinucleotide tandem repeats in humans and apes. J Mol Evol.

[B31] Stenson PD, Ball EV, Mort M, Phillips AD, Shiel JA, Thomas NS, Abeysinghe S, Krawczak M, Cooper DN (2003). Human Gene Mutation Database (HGMD): 2003 update. Hum Mutat.

[B32] Hamosh A, Scott AF, Amberger J, Bocchini C, Valle D, McKusick VA (2002). Online Mendelian Inheritance in Man (OMIM), a knowledgebase of human genes and genetic disorders. Nucleic Acids Res.

[B33] Xie D, Shu XO, Deng Z, Wen WQ, Creek KE, Dai Q, Gao YT, Jin F, Zheng W (2000). Population-based, case-control study of HER2 genetic polymorphism and breast cancer risk. J Natl Cancer Inst.

[B34] Healey CS, Dunning AM, Teare MD, Chase D, Parker L, Burn J, Chang-Claude J, Mannermaa A, Kataja V, Huntsman DG, Pharoah PD, Luben RN, Easton DF, Ponder BA (2000). A common variant in BRCA2 is associated with both breast cancer risk and prenatal viability. Nat Genet.

[B35] Soussi T (2000). The p53 tumor suppressor gene: from molecular biology to clinical investigation. Ann N Y Acad Sci.

[B36] Beckman G, Birgander R, Sjalander A, Saha N, Holmberg PA, Kivela A, Beckman L (1994). Is p53 polymorphism maintained by natural selection?. Hum Hered.

[B37] HapMap C (2003). The International HapMap Project. Nature.

[B38] Dumont P, Leu JI, Della Pietra AC, George DL, Murphy M (2003). The codon 72 polymorphic variants of p53 have markedly different apoptotic potential. Nat Genet.

[B39] Marin MC, Jost CA, Brooks LA, Irwin MS, O'Nions J, Tidy JA, James N, McGregor JM, Harwood CA, Yulug IG, Vousden KH, Allday MJ, Gusterson B, Ikawa S, Hinds PW, Crook T, Kaelin WGJ (2000). A common polymorphism acts as an intragenic modifier of mutant p53 behaviour. Nat Genet.

[B40] Storey A, Thomas M, Kalita A, Harwood C, Gardiol D, Mantovani F, Breuer J, Leigh IM, Matlashewski G, Banks L (1998). Role of a p53 polymorphism in the development of human papillomavirus-associated cancer. Nature.

[B41] Huttley GA, Easteal S, Southey MC, Tesoriero A, Giles GG, McCredie MR, Hopper JL, Venter DJ (2000). Adaptive evolution of the tumour suppressor BRCA1 in humans and chimpanzees. Australian Breast Cancer Family Study. Nat Genet.

[B42] Fleming MA, Potter JD, Ramirez CJ, Ostrander GK, Ostrander EA (2003). Understanding missense mutations in the BRCA1 gene: an evolutionary approach. Proc Natl Acad Sci U S A.

[B43] Pavlicek A, Noskov VN, Kouprina N, Barrett JC, Jurka J, Larionov V (2004). Evolution of the tumor suppressor BRCA1 locus in primates: implications for cancer predisposition. Hum Mol Genet.

[B44] Whitehouse C, Chambers J, Catteau A, Solomon E (2004). Brca1 expression is regulated by a bidirectional promoter that is shared by the Nbr1 gene in mouse. Gene.

[B45] Puente XS, Gutierrez-Fernandez A, Ordonez GR, Hillier LW, Lopez-Otin C (2005). Comparative genomic analysis of human and chimpanzee proteases. Genomics.

[B46] Angata T, Margulies EH, Green ED, Varki A (2004). Large-scale sequencing of the CD33-related Siglec gene cluster in five mammalian species reveals rapid evolution by multiple mechanisms. Proc Natl Acad Sci U S A.

[B47] Coffey DS (2001). Similarities of prostate and breast cancer: Evolution, diet, and estrogens. Urology.

[B48] Bingham S, Riboli E (2004). Diet and cancer--the European Prospective Investigation into Cancer and Nutrition. Nat Rev Cancer.

[B49] Poirier MC (2004). Chemical-induced DNA damage and human cancer risk. Nat Rev Cancer.

[B50] Finch CE, Stanford CB (2004). Meat-adaptive genes and the evolution of slower aging in humans. Q Rev Biol.

[B51] Enard W, Fassbender A, Model F, Adorjan P, Pääbo S, Olek A (2004). Differences in DNA methylation patterns between humans and chimpanzees. Curr Biol.

[B52] Kalnina Z, Zayakin P, Silina K, Line A (2005). Alterations of pre-mRNA splicing in cancer. Genes Chromosomes Cancer.

[B53] Balbín M, Fueyo A, Tester AM, Pendás AM, Pitiot AS, Astudillo A, Overall CM, Shapiro SD, López-Otín C (2003). Loss of collagenase-2 confers increased skin tumor susceptibility to male mice. Nat Genet.

[B54] Coussens LM, Werb Z (2002). Inflammation and cancer. Nature.

[B55] Balkwill F (2004). Cancer and the chemokine network. Nat Rev Cancer.

[B56] Vakkila J, Lotze MT (2004). Inflammation and necrosis promote tumour growth. Nat Rev Immunol.

[B57] King MC, Wilson AC (1975). Evolution at two levels in humans and chimpanzees. Science.

[B58] Cáceres M, Lachuer J, Zapala MA, Redmond JC, Kudo L, Geschwind DH, Lockhart DJ, Preuss TM, Barlow C (2003). Elevated gene expression levels distinguish human from non-human primate brains. Proc Natl Acad Sci U S A.

[B59] Enard W, Khaitovich P, Klose J, Zollner S, Heissig F, Giavalisco P, Nieselt-Struwe K, Muchmore E, Varki A, Ravid R, Doxiadis GM, Bontrop RE, Pääbo S (2002). Intra- and interspecific variation in primate gene expression patterns. Science.

[B60] Khaitovich P, Muetzel B, She X, Lachmann M, Hellmann I, Dietzsch J, Steigele S, Do HH, Weiss G, Enard W, Heissig F, Arendt T, Nieselt-Struwe K, Eichler EE, Pääbo S (2004). Regional patterns of gene expression in human and chimpanzee brains. Genome Res.

[B61] Bond GL, Hu W, Bond EE, Robins H, Lutzker SG, Arva NC, Bargonetti J, Bartel F, Taubert H, Wuerl P, Onel K, Yip L, Hwang SJ, Strong LC, Lozano G, Levine AJ (2004). A single nucleotide polymorphism in the MDM2 promoter attenuates the p53 tumor suppressor pathway and accelerates tumor formation in humans. Cell.

[B62] Matlashewski GJ, Tuck S, Pim D, Lamb P, Schneider J, Crawford LV (1987). Primary structure polymorphism at amino acid residue 72 of human p53. Mol Cell Biol.

[B63] Koushik A, Platt RW, Franco EL (2004). p53 codon 72 polymorphism and cervical neoplasia: a meta-analysis review. Cancer Epidemiol Biomarkers Prev.

[B64] Di Tommaso A, Soler C, Roos C, Kitzis A, Ladeveze V (2004). The ink4a/arf locus evolution in primates: characterization of three ARF sequences. DNA Cell Biol.

[B65] Zhang J, Rosenberg HF (2002). Diversifying selection of the tumor-growth promoter angiogenin in primate evolution. Mol Biol Evol.

[B66] Kent WJ (2002). BLAT--the BLAST-like alignment tool. Genome Res.

[B67] Altschul SF, Madden TL, Schaffer AA, Zhang J, Zhang Z, Miller W, Lipman DJ (1997). Gapped BLAST and PSI-BLAST: a new generation of protein database search programs. Nucleic Acids Res.

