# Supplementary material for: Comparative analysis of cancer genes in the human and chimpanzee genomes
Source: BMC Genomics. 2006 Jan 26;7:15. doi: 10.1186/1471-2164-7-15 (PMC1382208; doi:10.1186/1471-2164-7-15)
Supplement: Additional data file 2 — Chimpanzee cancer gene products identical to human orthologs. List of human genes identical to chimpanzee orthologs. Gene name, accession number, chromosomal location, protein length and functional category are indicated. [file 1471-2164-7-15-S2.doc]

**Table 1**

**Chimpanzee cancer gene products identical to human orthologs**

| **Name** | **Symbol** | **RefSeq** | **Locus** | **Lenght (aa)** | **Category** |
| --- | --- | --- | --- | --- | --- |
| B-cell CLL/lymphoma 10 | *BCL10* | NM_003921 | 1p22 | 233 | Apoptosis |
| damage-specific DNA binding protein 2 | *DDB2* | NM_000107 | 11p12 | 427 | DNA repair |
| MAD2 mitotic arrest deficient-like 1 | *MAD2L1* | NM_002358 | 4q27 | 205 | DNA repair |
| RAD51-like 1 | *RAD51L1* | NM_002877 | 14q23 | 350 | DNA repair |
| RAD9 homolog A | *RAD9A* | NM_004584 | 11q13 | 391 | DNA repair |
| GNAS complex locus | *GNAS* | NM_000516 | 20q13 | 394 | GTPase |
| v-Ha-ras Harvey rat sarcoma viral oncogene homolog | *HRAS* | NM_176795 | 11p15 | 170 | GTPase |
| v-Ki-ras2 Kirsten rat sarcoma viral oncogene homolog | *KRAS2* | NM_004985 | 12p12 | 188 | GTPase |
| neuroblastoma RAS viral oncogene homolog | *NRAS* | NM_002524 | 1p13 | 189 | GTPase |
| septin 5 | *SEPT5* | NM_002688 | 22q11 | 369 | GTPase |
| septin 6 | *SEPT6* | NM_015129 | Xq24 | 434 | GTPase |
| chimerin 1 | *CHN1* | NM_001822 | 2q31 | 459 | GTPase activator |
| Wiskott-Aldrich syndrome | *WAS* | NM_000377 | Xp11 | 502 | GTPase regulator |
| cyclin-dependent kinase 2 | *CDK2* | NM_001798 | 12q13 | 298 | Kinase |
| pim-1 oncogene | *PIM1* | NM_002648 | 6p21 | 313 | Kinase |
| protein kinase, cAMP-dependent, regulatory, type I, alpha | *PRKAR1A* | NM_002734 | 17q23 | 381 | Kinase |
| cyclin-dependent kinase inhibitor 1B | *CDKN1B* | NM_004064 | 12p13 | 198 | Kinase Inhibitor |
| cyclin-dependent kinase inhibitor 2A | *CDKN2A -p16INK4A* | NM_000077 | 9p21 | 156 | Kinase Inhibitor |
| succinate dehydrogenase complex, subunit C | *SDHC* | NM_003001 | 1q21 | 169 | Metabolism |
| nascent-polypeptide-associated complex alpha | *NACA* | NM_005594 | 12q23 | 215 | Nascent polypeptide associated complex |
| phosphatase and tensin homolog | *PTEN* | NM_000314 | 10q23 | 402 | Phosphatase |
| protein tyrosine phosphatase, non-receptor type 11 | *PTPN11* | NM_002834 | 12q24 | 593 | Phosphatase |
| FIP1 like 1 | *FIP1L1* | NM_030917 | 4q12 | 594 | Polyadenylation |
| cylindromatosis | *CYLD* | NM_015247 | 16q12 | 956 | Protease |
| ribosomal protein L22 | *RPL22* | NM_000983 | 3q26 | 128 | Ribosome |
| clathrin, heavy polypeptide | *CLTC* | NM_004859 | 17q11 | 1675 | Structural |
| RAB6 interacting protein 2 | *ELKS* | NM_015064 | 12p13 | 948 | Structural |
| growth arrest-specific 7 | *GAS7* | NM_003644 | 17p13 | 336 | Structural |
| LIM and SH3 protein 1 | *LASP1* | NM_006148 | 17q11 | 261 | Structural |
| lymphocyte cytosolic protein 1 | *LCP1* | NM_002298 | 13q14 | 627 | Structural |
| LIM domain containing preferred translocation partner | *LPP* | NM_005578 | 3q28 | 612 | Structural |
| moesin | *MSN* | NM_002444 | Xq11 | 577 | Structural |
| nucleophosmin | *NPM1* | NM_002520 | 5q35 | 294 | Structural |
| tropomyosin 4 | *TPM4* | NM_003290 | 19p13 | 248 | Structural |
| ZW10 homolog | *ZW10* | NM_004724 | 11q23 | 779 | Structural |
| topoisomerase I | *TOP1* | NM_003286 | 20q12 | 765 | Topoisomerase |
| paired related homeobox 1 | *PMX1* | NM_006902 | 1q24 | 217 | Transcription co-activator |
| B-cell CLL/lymphoma 11A | *BCL11A* | NM_018014 | 2p13 | 773 | Transcription Factor |
| runt-related transcription factor 1 | *CBFA2T1* | NM_004349 | 8q22 | 577 | Transcription Factor |
| v-ets erythroblastosis virus E26 oncogene like | *ERG* | NM_004449 | 21q22 | 462 | Transcription Factor |
| Friend leukemia virus integration 1 | *FLI1* | NM_002017 | 11q24 | 452 | Transcription Factor |
| GATA binding protein 1 | *GATA1* | NM_002049 | Xp11 | 413 | Transcription Factor |
| hepatic leukemia factor | *HLF* | NM_002126 | 17q22 | 295 | Transcription Factor |
| high mobility group AT-hook 2 | *HMGA2* | NM_003483 | 12q15 | 109 | Transcription Factor |
| homeo box A11 | *HOXA11* | NM_005523 | 7p15 | 313 | Transcription Factor |
| homeo box A13 | *HOXA13* | NM_000522 | 7p15 | 388 | Transcription Factor |
| homeo box C13 | *HOXC13* | NM_017410 | 12q13 | 330 | Transcription Factor |
| hyperparathyroidism 2 | *HRPT2* | NM_024529 | 1q21 | 531 | Transcription Factor |
| LIM domain only 1 | *LMO1* | NM_002315 | 11p15 | 156 | Transcription Factor |
| mothers against DPP homolog 4 | *MADH4* | NM_005359 | 18q21 | 552 | Transcription Factor |
| nuclear receptor coactivator 2 | *NCOA2* | NM_006540 | 8q13 | 1464 | Transcription Factor |
| paired box gene 5 | *PAX5* | NM_016734 | 9p13 | 391 | Transcription Factor |
| pre-B-cell leukemia transcription factor 1 | *PBX1* | NM_002585 | 1q23 | 430 | Transcription Factor |
| retinoic acid receptor, alpha | *RARA* | NM_000964 | 17q12 | 462 | Transcription Factor |
| T-cell acute lymphocytic leukemia 2 | *TAL2* | NM_005421 | 9q31 | 108 | Transcription Factor |
| T-cell leukemia, homeobox 3 | *TLX3* | NM_021025 | 5q35 | 291 | Transcription Factor |
| Ikaros | *ZNFN1A1* | NM_006060 | 7p12 | 519 | Transcription Factor |
| tripartite motif-containing 24 | *TIF1* | NM_003852 | 7q32 | 1016 | Transcription regulation |
| SMARC, subfamily b, member 1 | *SMARCB1* | NM_003073 | 22q11 | 385 | Tumor suppressor |
| suppressor of fused homolog | *SUFU* | NM_016169 | 10q24 | 484 | Tumor suppressor |
| F-box and WD-40 domain protein 7 | *FBXW7* | NM_018315 | 4q31 | 627 | Ubiquitylation |
| von Hippel-Lindau tumor suppressor | *VHL* | NM_000551 | 3p25 | 213 | Ubiquitylation |
| mixed-lineage leukemia translocated to, 11 | *AF1Q* | NM_006818 | 1q21 | 90 | Unknown |
| bromodomain containing 4 | *BRD4* | NM_014299 | 19p13 | 722 | Unknown |
| B-cell translocation gene 1 | *BTG1* | NM_001731 | 12q22 | 171 | Unknown |
| cysteine-rich hydrophobic domain 2 | *CHIC2* | NM_012110 | 4q11 | 165 | Unknown |
| papillary renal cell carcinoma | *PRCC* | NM_005973 | 1q21 | 491 | Unknown |
| PRO1073 protein | *PRO1073* | AF113016 | 11q31 | 57 | Unknown |
| Shwachman-Bodian-Diamond syndrome | *SBDS* | NM_016038 | 7q11 | 250 | Unknown |
| TRK-fused gene | *TFG* | NM_006070 | 3q11 | 400 | Unknown |
| TCF3 (E2A) fusion partner | *TFPT* | NM_013342 | 19q13 | 253 | Unknown |
